# Metabolic modelling reveals broad changes in gut microbial metabolism in inflammatory bowel disease patients with dysbiosis

**DOI:** 10.1038/s41540-021-00178-6

**Published:** 2021-05-06

**Authors:** Almut Heinken, Johannes Hertel, Ines Thiele

**Affiliations:** 1 School of Medicine, National University of Ireland, Galway, Ireland; 2 Ryan Institute, National University of Ireland, Galway, Ireland; 3 Department of Psychiatry and Psychotherapy, University Medicine Greifswald, Greifswald, Germany; 4 Division of Microbiology, National University of Galway, Galway, Ireland; 5 APC Microbiome Ireland, Cork, Ireland

**Keywords:** Microbiology, Biochemistry, Computer modelling

## Abstract

Inflammatory bowel diseases, such as Crohn’s Disease, are characterised by an altered blood and faecal metabolome, and changes in gut microbiome composition. Here, we present an efficient, scalable, tractable systems biology framework to mechanistically link microbial strains and faecal metabolites. We retrieve strain-level relative abundances from metagenomics data from a cohort of paediatric Crohn’s Disease patients with and without dysbiosis and healthy control children and construct and interrogate a personalised microbiome model for each sample. Predicted faecal secretion profiles and strain-level contributions to each metabolite vary broadly between healthy, dysbiotic, and non-dysbiotic microbiomes. The reduced microbial diversity in IBD results in reduced numbers of secreted metabolites, especially in sulfur metabolism. We demonstrate that increased potential to synthesise amino acids is linked to Proteobacteria contributions, in agreement with experimental observations. The established modelling framework yields testable hypotheses that may result in novel therapeutic and dietary interventions targeting the host-gut microbiome-diet axis.

## Introduction

The human gut microbiome plays an important role in human health and disease. It performs important functions, such as maturation of the host immune system, digestion of food, synthesis of short-chain fatty acids, vitamins, and amino acids, and protection against pathogens^1,2^. Changes in microbiome composition have been linked to complex multifactorial diseases, e.g., type 2 diabetes, metabolic syndrome, and non-alcoholic fatty liver syndrome^2,3^, as well as inflammatory bowel disease (IBD)^4^. IBD can be separated into two subtypes, Crohn’s Disease and ulcerative colitis^4^. Factors contributing to the IBD pathogenesis include genetics, diet, lifestyle, and the gut microbiome^5^. There is an urgent need for a mechanistic understanding of the role of these complex host-microbiome–environment interactions in IBD^6^. Ultimately, personalised treatment approaches targeting the diet-host-microbiome axis are needed^6^. A number of studies have reported differences in the abundances of certain taxa between IBD patients and healthy controls, identified through 16S rRNA sequencing or metagenomic approaches^6–8^. However, metagenomic approaches alone are insufficient to infer the functional metabolic activity of the microbiome^6^. Thus, functional, pathway-based analyses are required to elucidate not only the changes in composition in the gut microbiomes of IBD patients but also the metabolic changes that could serve as a target for therapeutic interventions.

Several studies have shown differences in the blood and/or faecal metabolome between IBD patients and cohorts^4^. Changes in microbial metabolites, such as short-chain fatty acids, secondary bile acids, and tryptophan, have been especially implicated in IBD^4^. To gain insight into changes in microbiome structure and function, metabolomic and metagenomic analyses for the same faecal samples are commonly performed, and subsequently, positive and negative correlations between species abundances and specific metabolites are inferred for cohorts of IBD patients and controls^9,10^. However, the mechanisms underlying these correlations remain unclear^9^ and it is difficult to disentangle the contributions of microbial and host metabolism to altered metabolite levels. Mechanism-based computational models that integrate omics data (e.g., metagenomics, metabolomics, and metatranscriptomics), as well as dietary information could mechanistically link changes in microbe abundances and metabolite levels and, ultimately, propose potential disease mechanisms, biomarkers, and personalised therapies^5^.

One such mechanistic modelling approach is constraint-based reconstruction and analysis (COBRA)^11^. Briefly, COBRA relies on a manually curated genome-scale reconstruction of metabolism of a target organism, which can be converted into a mathematical model and subsequently interrogated through simulations, using established methods, such as flux balance analysis^12^. COBRA models^13^ can be readily contextualised by implementing different types of data as constraints, e.g., metagenomics^14^, metabolomics^15^, proteomics^16^, or dietary information^17^. To enable constraint-based modelling that captures the diversity of the human gut microbiome, we have assembled a resource of 818 curated genome-scale reconstructions of human gut microbes, AGORA^18^. The reconstructions have been built on the strain level from refined genome annotations and experimental data^18^. AGORA enables the creation of personalised microbiome models from metagenomics data that freely allow host–microbe and microbe–microbe metabolic interactions^14^. Thus, these microbiome models have valuable applications in studying microbe–microbe and host–microbe interactions^19^. Several studies have already successfully applied AGORA to predict microbiome metabolism in IBD, such as predicting personalised dietary supplements^20^, predict the potential for metabolic cross-feeding were predicted in patients and controls^21^, stratify subtypes according to their metabolic networks^22^, and predict treatment efficacy^23^.

Previously, Lewis et al.^8^ have performed metagenomic sequencing of the microbiomes of paediatric Crohn’s Disease patients and healthy control children and found that the Crohn’s Disease microbiomes stratified into two clusters, i.e., a “near cluster”, which resembled the healthy microbiomes in composition and a “far cluster” characterised by microbial dysbiosis that was distinct in composition from both healthy controls and the near cluster microbiomes. Based on these results, they separated the patients into two groups, “non-dysbiotic” and “dysbiotic” IBD^8^. We have previously constructed personalised models for a subset of 20 dysbiotic Crohn’s Disease and 25 healthy control microbiomes from this cohort^24^. We have then applied the COBRA approach to predict the bile acid deconjugation and biotransformation potential of each sample^24^. The computational modelling stratified the Crohn’s Disease microbiomes and healthy microbiomes by their bile acid metabolism profiles^24^. Here, we substantially expanded the computation of metabolic profiles to a wide variety of metabolic subsystems. We systematically predicted the potential of each microbiome to secrete and take up all metabolites, for which biosynthesis pathways and transport reactions were present in the community models and identified the metabolites that best stratified the dysbiotic and non-dysbiotic individuals. For all secreted metabolites, we computed the respective contributing strains in each microbiome. Finally, we validated the predictions for amino acid metabolites against published metabolomic data from the same cohort. Taken together, we present a computational systems biology approach that bridges the gap between metagenomic and metabolomic data and provides mechanistic, testable hypotheses for metabolite–microbe associations.

## Results

**Fig. 1:**
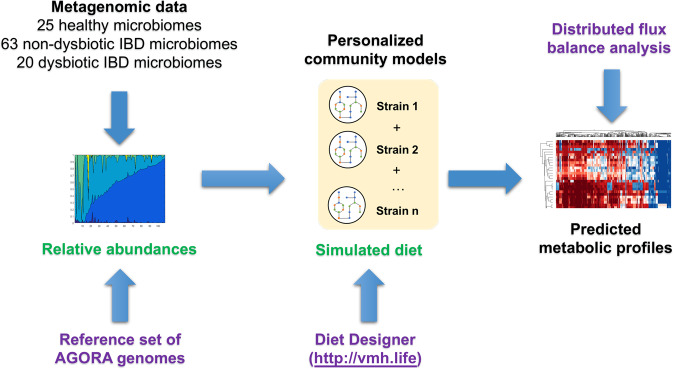
Schematic overview of the modelling framework established in this study. Metagenomic reads were mapped onto a reference set of AGORA^18^ genomes, and the strain-level relative abundances were retrieved. For each metagenomic sample, a personalised microbiome model was constructed. The models were parameterised further with a simulated “Average European” diet retrieved from the Virtual Metabolic Human^17^ database. The metabolic profile of each microbiome model was then computed with distributed flux balance analysis^53^. Required input data are shown in green and required tools are shown in purple.

In this study, we aimed at improving the understanding, which microbial metabolites may be altered in IBD due to altered faecal microbial community structure. Therefore, we predicted the metabolic profile, i.e., the combined quantitative potential of all community members to take up dietary metabolites and secrete metabolic end products, as well as the strain-level contributions to these overall fluxes, of 108 analysed microbiomes, corresponding to 20 IBD microbiomes with dysbiosis, 63 non-dysbiotic IBD microbiomes, and 25 control individuals as defined in ref. ^8^ (Fig. 1, Methods). We stratified the 108 microbiomes according to the predicted metabolic profiles. We demonstrate that the metabolite profiles of microbiomes from individuals with IBD and dysbiosis were distinct from both healthy microbiomes and IBD microbiomes with dysbiosis and identify the features that best separate these groups. Finally, we compared our results with published metabolomic data from the same individuals. In agreement with published findings, we predicted that dysbiotic Crohn’s Disease microbiomes have an increased potential to synthesise amino acids.

### Profiling the range of metabolic capabilities present in the 108 microbiomes

First, we developed a large-scale, computationally efficient constraint-based modelling approach (Fig. 1). Briefly, we created a personalised microbiome model for each sample by mapping the relative strain-level abundances onto the reference set of reconstructed organisms and by then joining the corresponding AGORA reconstructions together as described previously^24^. A community biomass reaction was formulated that ensured growth of the strains at experimentally determined ratios (Methods). Each personalised microbiome model was further contextualised by simulating the intake of an Average European diet (Methods). The metabolic profile of each microbiome was then computed as follows: All dietary, faecal, and strain-specific exchange reactions present in the model were retrieved, and the minimal and maximal fluxes through these exchange reactions were computed using distributed flux balance analysis^25^ (Methods). This approach enabled a systematic evaluation of secretion potential, uptake potential, and strain-specific contributions for each metabolite that could be transported by at least one microbiome model. The modelling framework captured the range of metabolic capabilities encoded by the human gut microbiome, and the variation in metabolic potential as a function of microbiome composition.

### Qualitative metabolic potential in the 108 microbiomes

Overall, the 108 microbiome models accounted for exchange reactions for 419 metabolites, for which the minimal and maximal fluxes were computed. We calculated the microbiomes’ theoretical potential to take up and secrete metabolites (Supplementary Table 1). Of the maximally possible 419 metabolites, 143 could be secreted into the faecal compartment by at least one microbiome but were not taken up, i.e., they were of microbial origin but not dietary. Another 59 metabolites could only be taken up by at least one microbiome and were thus only present in the simulated diet. Finally, further 86 metabolites were both taken up and secreted meaning they were both dietary and microbial of origin. The remaining 131 metabolites that could be neither taken up nor secreted either lacked the necessary biosynthesis precursors in the given dietary input or were dead-end metabolites, i.e., metabolites that only be consumed or produced, in the corresponding AGORA reconstructions. Some qualitative metabolite biosynthesis capacities were present in almost all microbiomes metabolite while others were rare (Supplementary Table 1). Taken together, the systematic in silico metabolic profiling predicted that the analysed microbiomes could convert the dietary inputs into a variety of metabolites from diverse subsystems.

### Distinct metabolite uptake and secretion potential in dysbiotic compared with non-dysbiotic microbiomes

Since the microbial composition of the dysbiotic and non-dysbiotic individuals clearly differed^8^, we expected these microbial differences to be reflected in the predicted metabolic profiles. We performed a statistical analysis (Methods) of the predicted uptake and secretion fluxes reported in Supplementary Tables 2–3. Of the 229 metabolites that were produced by at least one microbiome, 44 differed statistically significantly between the microbiomes of IBD patients and healthy controls (Wilcoxon rank sum test corrected for false discovery rate, Supplementary Table 4a). Moreover, 122 metabolites had statistically significantly different production potential in the dysbiotic compared with the non-dysbiotic IBD cluster (Supplementary Table 4b). Overall, the production potential for 139 metabolites differed between at least two of the three groups (Supplementary Table 4a, b). The clearer separation between the dysbiotic and non-dysbiotic IBD cluster than between healthy and IBD was in line with our expectations as the modelling framework was personalised with only the individuals’ gut microbial compositions and did not account for other factors (e.g., human metabolism and non-microbiome-mediated effects of medication). Taken together, dysbiotic IBD microbiomes were distinct in metabolite secretion potential as a direct consequence of their distinct microbial compositions.

**Fig. 2:**
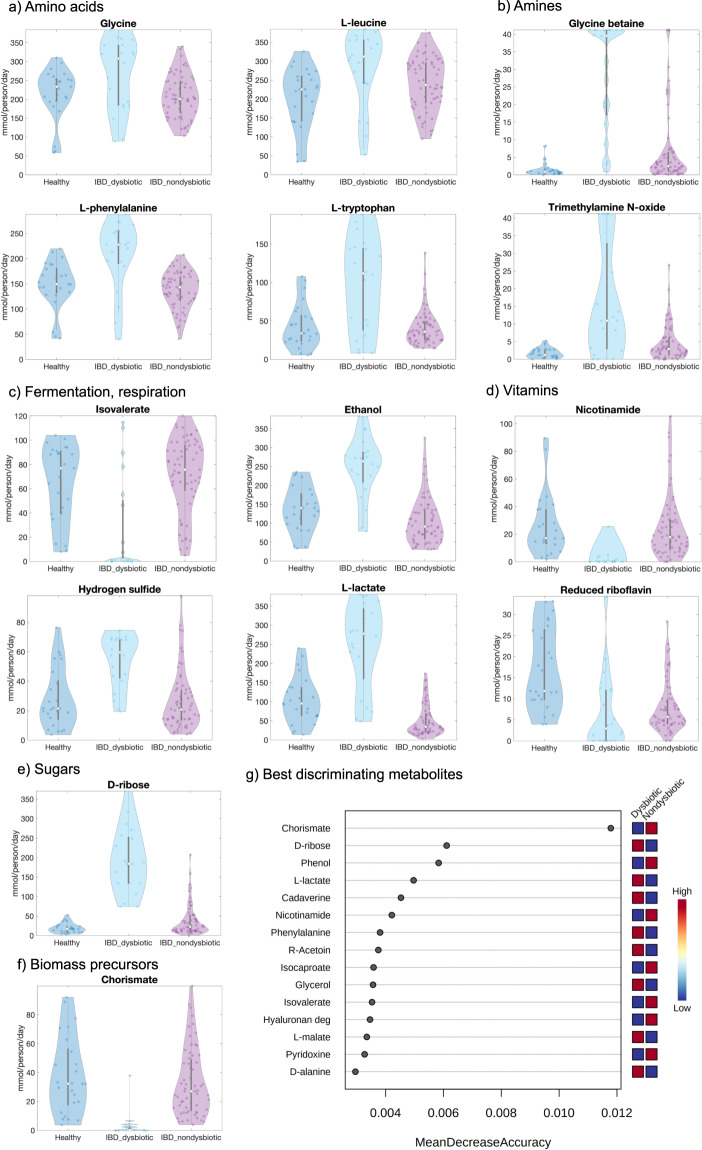
Predicted metabolite secretion profiles for the 108 microbiomes. **a**–**f** Total metabolite secretion fluxes (mmol/person/day) predicted for the microbiomes of 20 paediatric Crohn’s Disease patients with dysbiosis (IBD_dysbiotic), 63 paediatric Crohn’s Disease patients without dysbiosis (IBD_nondysbiotic), and 25 healthy control children (Healthy) for selected metabolites of interest. **g** Random forests analysis showing the secreted metabolites that best stratified the 20 dysbiotic and 63 dysbiotic IBD microbiomes ranked by their contributions to classification accuracy (represented by the variable MeanDecreaseAccuracy).

The dysbiotic IBD microbiomes were depleted in the production potential for metabolites involved in glycan degradation, fermentation, and B-vitamin biosynthesis (Fig. 2a–f). Metabolites with increased production potential in the dysbiotic cluster belonged mainly to the subsystems of amino acid metabolism, TCA cycle, simple sugars, and lipid metabolism (Fig. 2a–f). For instance, we predicted an increased secretion potential in the dysbiotic cluster for lactate, glycine, betaine, hydrogen sulfide, ethanol, putrescine, and trimethylamine N-oxide (TMAO) (Fig. 2a–f and Supplementary Table 4b). Increased faecal lactate^9^ and putrescine^26^ have been reported for Crohn’s Disease patients. Hydrogen sulfide has been proposed to both worsen^27^ and protect against^28^ gastrointestinal inflammation. On the other hand, a reduced secretion potential was predicted for branched-chain fatty acids, nicotinamide (vitamin B3), and degradation products of mucins and other glycans (Fig. 2a–f and Supplementary Table 4b). Reduced faecal isovalerate has been observed in IBD^26^. Decreased vitamin B3 levels have also been reported for IBD patients^9,10,26^. Riboflavin and reduced riboflavin biosynthesis were decreased in IBD microbiomes compared with healthy (Fig. 2d and Supplementary Table 4a). *Faecalibacterium prausnitzii*, which is well known to be depleted in Crohn’s Disease patients^29^, uses riboflavin as a redox mediator in an extracellular electron shuttle^30^ explaining the decreased riboflavin reduction. A Random forests analysis was performed on the metabolite secretion fluxes in MetaboAnalyst^31^ with an out-of-bag (OOB) error of 0.0648. Among the secreted metabolites that best stratified the non-dysbiotic and dysbiotic microbiomes were chorismate, d-ribose, l-lactate, and phenol (Fig. 2g).

**Fig. 3:**
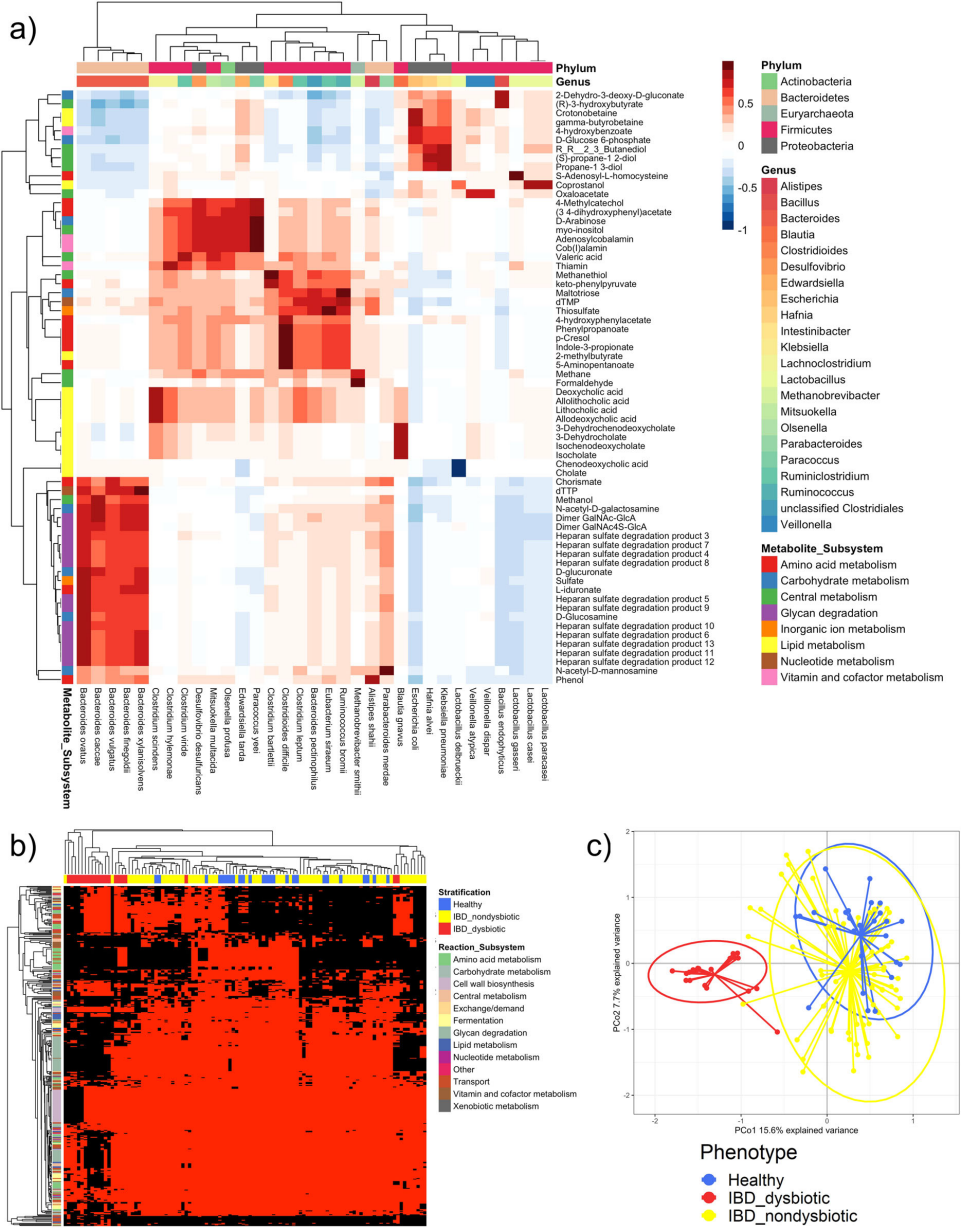
Metabolic properties and flux profiles computed for the 108 microbiomes. **a** Spearman correlations between metabolite secretion fluxes (mmol/person/day) and species-level relative abundances across all 108 microbiome models. Shown are only the 66 metabolites for which the positive or negative correlation with at least one species was higher than 0.75 or lower than −0.75, respectively. Rows show metabolites annotated by subsystem, and columns show species annotated by genus and phylum. **b** Absolute reaction presence in the 108 microbiome models than differed significantly (*p*-value corrected for false discovery rate < 0.05, Supplementary Table 4b) between the 20 dysbiotic and 63 dysbiotic IBD microbiomes. Rows show reactions annotated by subsystem, and columns show microbiome models annotated by group. Red = reaction present, black = reaction absent. **c** Principal coordinates analysis of all 26,873 strain to metabolite contributions (mmol/person/day, Supplementary Table 6) computed for the 108 microbiome models.

To link the predicted metabolite secretion potential to specific microbes, we calculated the Spearman correlation between metabolite secretion and uptake potential and species abundances. Strong correlations (>0.75) between species and metabolites were found for 66 secreted metabolites (Fig. 3a). For instance, glycan degradation products strongly correlated with several *Bacteroides* spp., in agreement with *Bacteroides* being known glycan degraders^32^ (Fig. 3a). Secondary bile acids correlated with species known to biotransform bile acids (Fig. 3a) as observed previously^24^. As expected, methane correlated positively with *Methanobrevibacter smithii*^33^, and p-cresol correlated with the known p-cresol producer *Clostridioides difficile*^34^ (Fig. 3a). The remaining 163 secreted metabolites did not strongly correlate with specific species. Thus, the uptake and production of these metabolites was carried out by a combination of multiple taxa.

### Absolute and quantitative presence of metabolic functions is altered in dysbiotic microbiomes

To explain the observed changes in metabolite secretion potential, we calculated and inspected the absolute presence of reactions in the microbiomes, as well as the quantitative reaction abundances on the whole community, class, and genus level. The absolute presence of 84 and 393 reactions was distinct between healthy and IBD and between the dysbiotic and non-dysbiotic IBD cluster, respectively (Fig. 3b and Supplementary Table 4b). Thus, the dysbiotic IBD microbiomes were depleted or enriched in the absolute presence of certain pathways. Specifically, reactions involved in glycan degradation were absent in most dysbiotic microbiomes (Fig. 3b).

The abundances of 460 reactions on the total community level, 868 reactions on the phylum level, and 37,059 reactions on the genus level differed significantly between healthy controls and IBD patients (Supplementary Table 4a). Moreover, 1397 reactions on the total community level, 5443 reactions on the phylum level, and 49,376 reactions on the genus level were different in abundance between the dysbiotic and non-dysbiotic IBD cluster (Supplementary Table 4b). The abundances of complete pathways were distinct between groups. For instance, amino acid biosynthesis and lipid metabolism pathways had higher abundances in the dysbiotic cluster (Supplementary Fig. 1). Taken together, the microbiomes of dysbiotic IBD patients were distinct in the qualitative and quantitative presence of key reactions and pathways, which explains their aforementioned altered potential to consume and secrete metabolites.

### IBD microbiomes exhibit reduced metabolic diversity and altered sulfur secretion patterns

**Fig. 4:**
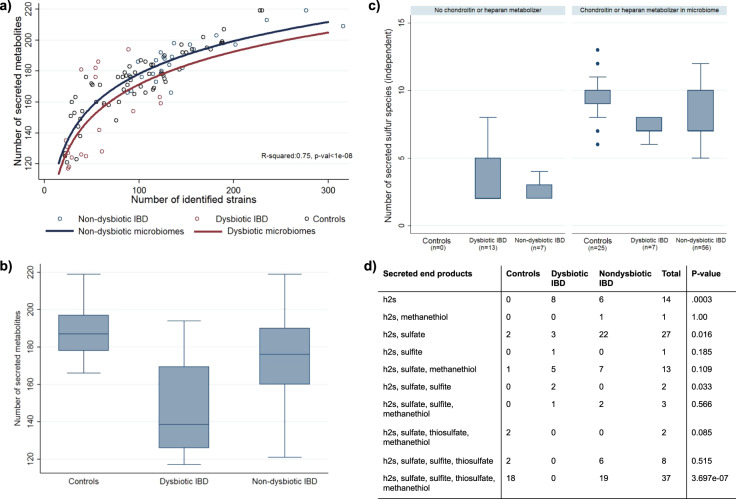
Reduced metabolic diversity in dysbiotic IBD. **a** Logarithmic dependency of the number of secreted metabolites in dependency on the number of identified strains. Curves for dysbiotic (red) and non-dysbiotic (blue) microbiomes are statistically different (*b* = −6.81, 95%−CI:(−13.38;−0.25), *p* = 0.042). **b** Box plots for the number of secreted metabolites for the three study groups. Differences across the three study groups are significant (*p* < 1e-08). **c** Number of secreted not perfectly coupled sulfur species in dependency on study group and the presence of glycan-degrading species. The presence of glycan producing species mediates significantly the effects of the study group (mediation effect: 53.11%, 95%−CI:(28,64%;77.60%, *p* = 2.14e-05). **d** Distribution of secretion patterns of end products of bacterial sulfur metabolism across the study groups. Abbreviations for metabolites are from https://www.vmh.life. *p*-values were derived from Fisher’s exact test. In the box plots, the centre lines represent the median; box limits represent upper and lower quartiles; whiskers represent 1.5x interquartile range.

We hypothesised that the reduced species diversity in the IBD microbiomes with dysbiosis^8^ would also translate into reduced metabolic diversity and in a reduced number of secreted compounds. Thus, we further analysed the qualitative metabolic potential per microbiome discussed above. Indeed, the number of compounds that could be theoretically secreted by each microbiome was highly dependent on the number of strains found in the stool samples, showing a logarithmic dependency (Fig. 4a). Additionally, the metabolic diversity in dysbiotic IBD was more restricted as predicted from the loss in microbial diversity alone (*b* = −6.81, 95%CI:(−13.38;−0.25), *t*(105) = −2.06, *p* = 0.042) (Fig. 4a, b).

Next, we analysed the number of secreted sulfur-containing metabolites, as import breakdown products from microbial sulfur metabolism (e.g., hydrogen sulfide) have been repeatedly implicated in the pathophysiology of IBD^27,35,36^. While taurine, hydrogen sulfide, methionine, and cysteine could be secreted by all microbiomes, the capability to produce other sulfur species, such as thiosulfate, sulfite, and sulfate, differed strongly between the three study groups (Supplementary Table 5). Mirroring the results above, the number of sulfur species that could theoretically be produced was drastically reduced in dysbiotic IBD microbiomes in comparison to healthy controls (Fig. 4c). From the 11 independent sulfur species tested, eight showed significantly increased likelihood (FDR < 0.05) to be present in the secretion profiles of healthy. Accordingly, the number of statistical independent sulfur species (e.g., not being perfectly correlated in their occurrence) was classifying healthy controls from dysbiotic IBD cases nearly perfectly (AUC = 0.95, *p* = 0.002, Supplementary Fig. 2). Interestingly, non-dysbiotic microbiomes still showed reduced capability to produce a variety of sulfur species (*p* = 0.003), revealing that also non-dysbiotic IBD microbiomes displayed an altered sulfur metabolism pattern. Consequently, when analysing the pattern of final breakdown products prevalent in secretion profiles, we found different distributions for these patterns across the three study groups (Fig. 4d).

Investigating species that could be causal to this loss in diversity in sulfur metabolites, we noticed that the species capable of using host glycans, i.e., chondroitin sulfate and heparan sulfate, as carbon source were depleted in most dysbiotic communities. Consequently, the associated glycan degradation reactions were also absent in these microbiomes (Fig. 3b). Thus, we followed the hypothesis that breakdown products of heparan and chondroitin may change systematically the availability of sulfur-containing metabolites in microbiomes, increasing therefore the metabolic diversity. Indeed, the presence of any heparan and chondroitin degrading species was statistically mediating the effect of IBD on the diversity in sulfur metabolism (mediation effect: 53.11%, 95%-CI:(28,64%;77.60%), *z* = 4.29, *p* = 1.79e-05). Therefore, over 50% of the difference in number of secreted sulfur compounds between healthy and dysbiotic IBD communities could be attributed to the lack of heparan and chondroitin degrading species in IBD. Consequently, IBD microbiomes containing *any* heparan and chondroitin degrading species were more similar to healthy controls in terms of diversity in sulfur metabolism (Fig. 4c).

Concluding, community modelling delivers a methodology to investigate the metabolic diversity of microbial communities. We found that metabolic diversity is reduced in IBD, in particular in sulfur metabolism. We delivered evidence that the loss of chondroitin sulfate and heparan sulfate degrading species may be causal to the loss in metabolic diversity. These species should be therefore considered as corner-stone species within the human microbiome.

### A wide variety of taxon to metabolite contributions are altered in IBD microbiomes

Faecal gut metabolite levels are altered in IBD patients, including many host-microbial co-metabolites, however, the contributions of specific microbes to these changes are often unknown^9^. To gain insight into which microbial taxa are responsible for the altered metabolic profiles in dysbiotic IBD patients, we modelled the strain-to-metabolite contributions directly by predicting the quantitative contribution of each strain to each secreted metabolite in each individual microbiome.

All 601 strains present in at least one microbiome contributed to the secretion of at least one metabolite indicating they should be able to potentially influence host metabolism. In total, 26,873 strain-to-metabolite contributions were predicted, corresponding on average to 44.71 contributions per strain (Supplementary Table 6). Of the 26,873 strain-to-metabolite contributions, 2609 (9.71%) and 7828 (29.13%) were statistically significantly different between healthy and IBD (Supplementary Table 4a), and dysbiotic and non-dysbiotic IBD (Supplementary Table 4b), microbiomes, respectively. Hence, a wide variety of metabolic fluxes was distinct in dysbiotic microbiomes. A principal coordinates analysis of all 26,873 strain-to-metabolite demonstrated that the dysbiotic IBD microbiomes clustered separately from both the healthy and the non-dysbiotic IBD microbiomes (Fig. 3c). Again, this separation was expected as the models directly reflected the distinct microbiome compositions of the dysbiotic IBD patients.

**Fig. 5:**
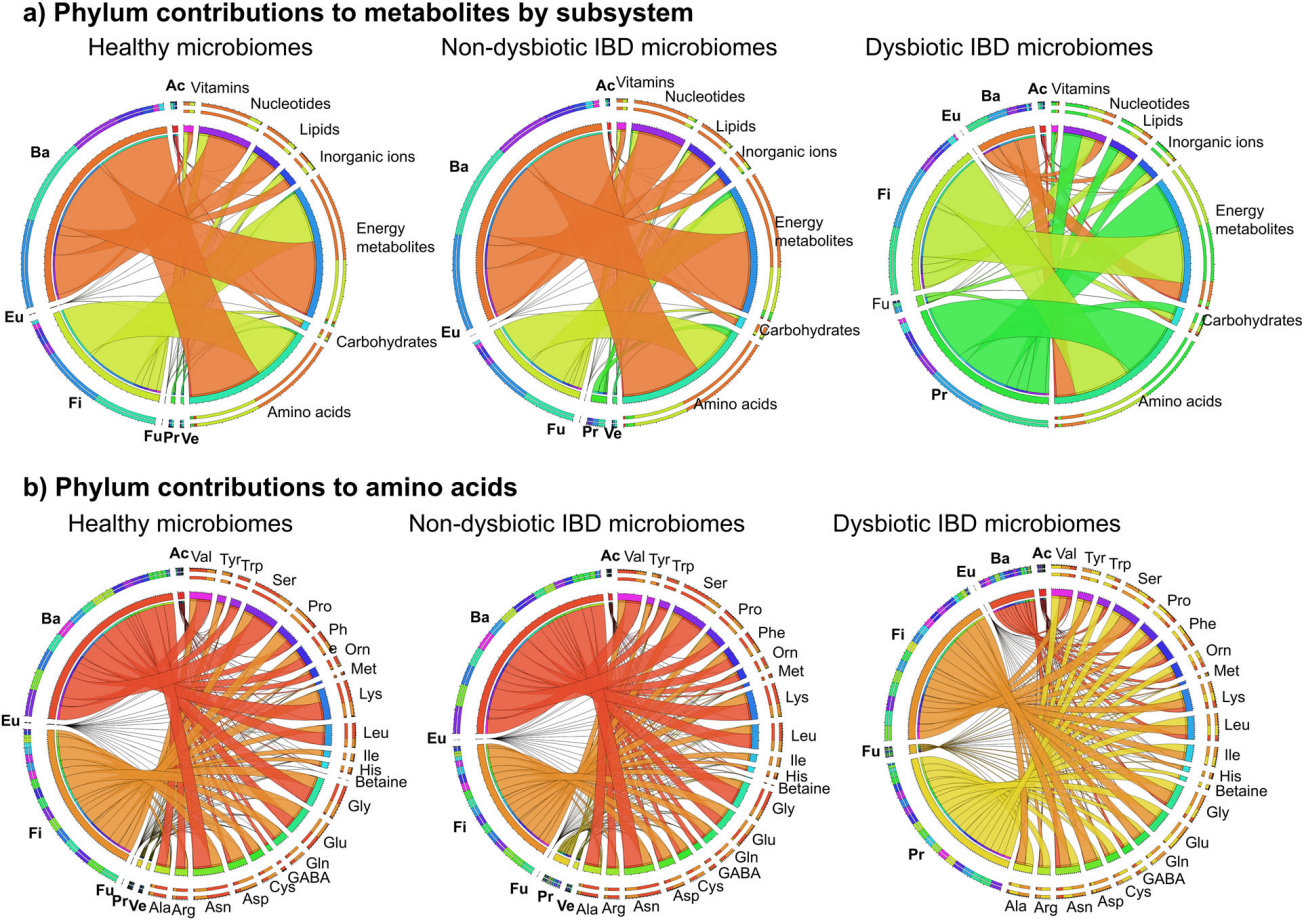
Strain to metabolite contributions computed for the three clusters summarised by the seven most prominent phyla. Shown are the contributions (mmol/person/day, Supplementary Table 6) summarised separately for the 25 healthy, 63 non-dysbiotic IBD, and 20 dysbiotic IBD microbiomes. **a** Phylum-level contributions to all metabolites summarised by metabolite subsystem. **b** Phylum-level contributions to 22 amino acid metabolites. Ac = Actinobacteria, Ba = Bacteroidetes, Eu = Eurarchaeota, Fi = Firmicutes, Fu = Fusobacteria, Pr = Proteobacteria, Ve = Verrucomicrobia. Phyla that did not contribute in a given cluster are omitted for that cluster.

Next, we investigated the contributions that were distinct in dysbiotic microbiomes by taxon. The contributions were summarised by phylum and by metabolite subsystem for the three clusters (Fig. 5a). The contribution flux profiles in the dysbiotic cluster clearly differed from both the healthy and non-dysbiotic IBD cluster, showing a drastic reduction in Bacteroidetes contributions accompanied by an increase in Proteobacteria and Fusobacteria contributions (Fig. 5a). This result is in line with observations that the dysbiotic cluster was characterised by an increase in gammaproteobacterial genera, and a corresponding decrease in Bacteroidetes and Clostridia^8^. While the non-dysbiotic cluster’s phylum to subsystem contributions resembled overall the profile of the healthy cluster, there was a slight increase in Fusobacteria and Proteobacteria contributions compared with the latter (Fig. 5a). This change may indicate that the individuals in the non-dysbiotic IBD cluster represent an early state in the development towards pronounced dysbiosis as observed in the dysbiotic cluster. The altered contribution profiles in the dysbiotic cluster were observed across all metabolic subsystems (Fig. 4a) demonstrating a broad effect of microbial composition changes on metabolite fluxes.

To gain more insight into metabolites of interest that were enriched or depleted in the dysbiotic cluster, we extracted the strain contributions (Supplementary Table 6) by metabolite. For instance, not only the total butyrate production flux was reduced in dysbiotic IBD microbiomes (Fig. 2a), but the contributing strains also differed between dysbiotic and non-dysbiotic microbiomes (Supplementary Fig. 3). As expected, known butyrate producers, e.g., *Roseburia* spp., *Faecalibacterium praunitzii*, and *Eubacterium rectale*, contributed the majority of butyrate in healthy and non-dysbiotic IBD microbiomes, while in dysbiotic microbiomes, butyrate secretion by these species was reduced (Supplementary Fig. 3). Reduced butyrate levels in IBD patients with dysbiosis have previously been attributed to reduced abundance of *Roseburia* and *Faecalibacterium* spp.^26,37^. For l-lactate, hydrogen sulfide, and TMAO, the total production potential was increased in dysbiotic microbiomes (Fig. 2c), and production of these compounds could mainly be attributed to Gammaproteobacteria genera, e.g., *Escherichia* spp., *Klebsiella* spp., *Enterobacter* spp., and *Sutterella* spp. (Supplementary Fig. 4–6). On the other hand, nicotinamide, which was clearly reduced in the dysbiotic cluster (Fig. 2d), was synthesised by representatives of the Bacteroidia class. (Supplementary Fig. 7). Thus, through metabolic modelling, we could retrieve a strain and individual-resolved, detailed snapshot of each metabolite of interest. Butyrate, l-lactate, TMAO, and hydrogen sulfide were produced by a combination of multiple species (Supplementary Figs. 4–6), explaining why no strong correlations with any individual species were observed for these metabolites. This result highlights that species-metabolite links may be missed in correlation-based approaches and demonstrates the added value of strain-and molecule-resolved simulations.

To summarise, we systematically interrogated the gut microbe-metabolite axis through constraint-based modelling. For each metabolite, the exact contributing strains were identified (Supplementary Figs. 3–7 and Supplementary Table 6). Overall, commensal and beneficial taxa contributed more to metabolites that are thought to be relevant for health (e.g., butyrate, B-vitamins), and taxa associated with dysbiosis (i.e., Proteobacteria, Bacilli) contributed more to potentially harmful metabolites, such as lactate, hydrogen sulfide, and TMAO.

### Modelling provides evidence for increased proteobacterial amino acid biosynthesis potential in dysbiosis

It has been proposed that metabolic modelling could be used to link metagenomic and metabolomic findings^38^. Previously, faecal metabolomic profiles were for amino acids determined^39^ for the same cohort^8^ that we used in this study. The patients in the dysbiotic cluster had distinct faecal metabolomic profiles, which have been characterised by an increase in amino acids and amino acid derivatives^39^. Of the faecal amino acid metabolites measured metabolomically by Ni et al., 23 overlapped with metabolites secreted by the microbiome models (Supplementary Table 7). When comparing significant differences between the non-dysbiotic and the dysbiotic IBD cluster in the metabolomic data and in silico, findings agreed in 16 cases and disagreed in 7 cases (Supplementary Table 7), which refers to an agreement in 69.6%. However, this agreement failed narrowly to be significantly different from an agreement by chance (Fisher’s exact test: *p* = 0.086), and future studies with bigger sample size are needed to corroborate the effectiveness of COBRA modelling to predict metabolomic changes in IBD. In agreements with metabolomic findings, we predicted an increased potential to synthesise betaine, glutamate, glycine, leucine, phenylalanine, and tryptophan in the dysbiotic IBD cluster (Fig. 2a, b and Supplementary Table 4b). The model additionally predicted that aspartate, GABA, isoleucine, and tyrosine were higher in the dysbiotic cluster (Supplementary Table 7). While faecal metabolomic concentrations of these amino acids did not reach significance when comparing between the non-dysbiotic and dysbiotic cluster, they were significantly different between healthy controls and IBD cases^39^. Note that it was not possible to compare secretion fluxes with the raw metabolomic data as the latter was not available.

Ni et al.^39^ also reported that faecal amino acid concentrations correlated with the abundance of Proteobacteria species and with the severity of the disease. Based on these findings, they proposed that increased proteobacterial utilisation of nitrogen for amino acid biosynthesis plays a role in the development of dysbiosis and Crohn’s Disease^39^. We computed the quantitative microbial contributions to the 23 amino acid metabolites that overlapped between in silico computed metabolites in this study and experimentally measured metabolites^39^. The proteobacterial contributions to amino acid metabolites were indeed substantially increased in the dysbiotic cluster (Fig. 5b). In contrast, in the healthy controls and the non-dysbiotic IBD cluster, amino acids were mainly synthesised by Bacteroidetes and Firmicutes representatives (Fig. 5b). Next, the detailed contribution profiles on the strain level were extracted for the examples of glycine, phenylalanine, leucine, tyrosine, and tryptophan (Supplementary Figs. 8–12). In the healthy controls and in the non-dysbiotic cluster, these amino acids were mainly synthesised by commensal genera, such as *Alistipes*, *Bacteroides*, *Faecalibacterium*, and *Roseburia* spp. (Supplementary Figs. 8–12). In contrast, the dysbiotic cluster was enriched in contributions by opportunistic pathogens, such as *Bacteroides fragilis*, *Escherichia*, *Haemophilus*, *Klebsiella* and *Streptococcus* spp. (Supplementary Figs. 8–12). In summary, modelling revealed an increased biosynthesis potential for amino acids and increased proteobacterial contributions to amino acids in the microbiomes of patients with dysbiosis, in agreement with the findings of Ni et al.^39^.

## Discussion

We have systematically profiled the metabolic potential of 108 individual microbiomes in silico. The metabolic profiles varied greatly across individual microbiomes reflecting the variation in microbial composition. We determined the net production and uptake potential of each microbiome, the qualitative and quantitative presence of reactions and pathways in each microbiome, the correlations between net production potential and reaction abundance, and finally the quantitative contributions of all strains present in the microbiomes to all secreted metabolites.

A wide variety of metabolic network properties and fluxes differed between dysbiotic IBD microbiomes and non-dysbiotic microbiomes (Supplementary Table 4b). First, the qualitative and quantitative presence of reactions and pathways differed in dysbiotic microbiomes (Fig. 3b and Supplementary Table 4b). As a result of their disturbed metabolic network structure, dysbiotic microbiomes also demonstrated an altered potential to take up and secrete metabolites (Fig. 2 and Supplementary Table 4b). The quantitative contributions of each strain to each secreted metabolite differed clearly between non-dysbiotic and dysbiotic microbiomes (Figs. 3c and 4; Supplementary Table 4b). This constraint-based modelling framework enabled us link microbes and metabolites in the context of the gut microbial community resulting in detailed biosynthesis profiles of each metabolite (Supplementary Figs. 3–12). We confirmed known microbe-metabolite links, e.g., butyrate production of *Faecalibacterium* and *Roseburia* spp.^37^ (Supplementary Fig. 3), and lactate production of *Lactobacillus*, *Streptococcus*, and *Escherichia* spp. (Supplementary Fig. 4). In addition, we propose contributing microbes for less-studied metabolites. For instance, gut microbes regulate circulating tryptophan levels and impaired tryptophan metabolism may play a role in IBD^40^. We here predict that, e.g., *Bacteroides*, *Coprococcus*, and *Odoribacter* spp. can synthesise tryptophan (Supplementary Fig. 11) and thus may play a role in mediating host–microbe interactions in tryptophan metabolism.

Importantly, our results provide clear evidence that reduced microbial diversity in strains leads to reduced metabolic diversity (e.g., reduced number of secreted metabolites). In the case of IBD, this includes particularly microbial sulfur metabolism with potential clinical consequences. Following a stoichiometric argument, all sulfur consumed by microbial communities must be released either by death or secretion. Assuming that the uptake of sulfur is relatively constant across microbiomes, reduced numbers in secreted sulfur metabolites must result in higher secretion potentials for the few secreted compounds. In accordance, the ubiquitously secreted hydrogen sulfide showed higher secretion potentials in dysbiotic IBD microbial communities, which displayed drastically reduced numbers in secreted sulfur compounds. Hydrogen sulfide is considered to be pro-inflammatory in the gastrointestinal environment^27,35,36^. Subsequently, restoring species diversity may reduce the potentially harmful production of hydrogen sulfide. Interestingly, a recent study has identified a link between changes in gut microbial sulfur metabolism and Crohn’s Disease^41^.

Importantly, the loss in diversity in sulfur metabolism was mediated by the loss of glycan-degrading species. Host glycan degradation is mainly attributed to *Bacteroides* spp.^32^, the abundances of which strongly correlated with glycan degradation product secretion in our study (Fig. 3a). In this regard, we note that the oral administration of chondroitin sulfate was already shown to be effective in IBD in a small clinical trial^42^. While the authors of the study attributed this effect to the general anti-inflammatory properties of chondroitin sulfate^42,43^, one may speculate that chondroitin sulfate is a prebiotic, promoting the growth of glycan-degrading species. Thus, promoting glycan degraders may improve species diversity, subsequently metabolic diversity, and may decrease by proxy the production of harmful microbial metabolites (e.g., hydrogen sulfide). A previous metabolic modelling approach already predicted dietary metabolites that could improve short-chain fatty acid secretion profiles in IBD^20^. Here, we additionally propose a mechanistic explanation for potentially beneficial dietary interventions. In conclusion, by computational modelling of microbial communities, we identified a potentially beneficial intervention on gut microbiota in IBD, which could be applied in a personalised manner in IBD cases specifically lacking glycan-degrading species. This underlines the utility of analysing the metabolic capabilities of microbial communities with mechanistic, strain-and molecule-resolved modelling tools.

Through computational modelling of IBD microbiomes, we could contextualise metabolomic findings^39^ from the same cohort. While one cannot compare fluxes and concentrations^44^, the trends of upregulated and downregulated metabolites in the dysbiotic cluster can nonetheless be compared. Indeed, for 16 of 23 amino acid metabolites, in silico fluxes agreed with measured faecal concentrations (Supplementary Table 7). While this agreement narrowly failed to be significant, it points towards the utility of COBRA modelling in predicting real metabolomic changes in IBD. Larger samples are needed to corroborate these results. Note that the ability to predict in vivo changes is dependent on the sample size utilised to reveal significant in vivo changes. In fact, in a similar constraint-based modelling approach using data from over 600 colorectal cancer patients and controls, good agreement between model predictions and faecal metabolomic measurements has been found^45^.

Importantly, the microbiome models used in our modelling framework only reflected individual microbiomes’ metabolic capabilities and did not account for host metabolism, moreover, they were not tailored towards the metabolomic data. Thus, the computed fluxes emerged entirely as a function of individual-specific microbiome structure and function. Hence, disagreements between trends in in silico fluxes and faecal metabolomic concentrations (Supplementary Table 7) do not necessarily reflect shortcomings of the modelling but instead point towards additional influences of human metabolism and/or diet on the seven amino acid metabolites, for which the model disagreed with the reported faecal concentrations. Owing to the ability of the modelling to disentangle microbial influences on faecal metabolites from human metabolism and diet, the modelling also provides evidence that the increased faecal amino acid concentrations measured by Ni et al.^39^ are at least in part due to the gut microbiome rather than only of dietary origin. Hence, the modelling confirmed the proposed link between increased abundances of Gammaproteobacteria and increased faecal amino acid levels.

Taken together, the present study demonstrates that an integrative, scalable constraint-based modelling framework enables the comprehensive characterisation of personalised gut microbiome models and the stratification of individuals based on flux profiles. Previous studies have already demonstrated that personalised models built using the AGORA models could stratify the microbiomes of IBD patients and controls^20,21,23^, and of subtypes of IBD^22^, according to their metabolic networks. In this study, we demonstrate that microbiome modelling can additionally predict the sample-contributions of each microbe to each metabolite. Moreover, we validated our results against metabolomic data from the same cohort. As a drawback of the present study, the raw metabolomic data was not available and could not be compared on an individual sample level with fluxes. Another study recently demonstrated that fluxes generated through the same microbiome modelling approach could be directly correlated with raw faecal metabolomic data and showed a very good agreement^45^.

While our results provide additional evidence that the gut microbiome influences the faecal metabolome, it is also influenced by host metabolism and diet. We have recently demonstrated that microbiome models as well as dietary information and physiological data can be integrated with a whole-body metabolic model of human, enabling personalised, organ-resolved predictions of host metabolic states^46^. The whole-body human model also allows the personalised prediction of the urinary, blood and serum metabolomes^46^, which have been reported to be altered in IBD patients^47–50^. Integrated host-microbiome metabolic modelling will allow the personalised prediction of these host blood, urine, and tissue metabolomes as a function of dietary input and microbial activity. Ultimately, an iterative pipeline of computational predictions and experimental validation may yield in the discovery of novel therapeutic and dietary interventions targeting the host-gut microbiome-diet axis.

## Methods

### Creation of personalised models

Paired end Illumina raw reads of 83 IBD patients in the PLEASE cohort^8^ and of 25 healthy controls in the COMBO cohort^51^ had been previously retrieved from NCBI SRA under SRA: SRP057027^20^. The reads had been preprocessed and mapped onto the reference set of AGORA genomes^20^. Publicly available metadata for the samples was retrieved from https://github.com/chvlyl/PLEASE and the sample stratification into the groups control, cluster 1 (non-dysbiotic IBD), and cluster 2 (dysbiotic IBD) was adapted as defined by the original authors^8^.

Personalised models for the 108 samples were created using Version 1.03 (published on 25.02.2019, available at https://www.vmh.life) of the AGORA resource^18^. To build the personalised models, the COBRA Toolbox^52^ extension Microbiome Modelling Toolbox^14^ was used. Personalised microbiome models were created in MATLAB (Mathworks, Inc.) version R2018b using the mgPipe module, as described previously^24^. Each personalised model contained a community biomass reaction, which was parameterised by applying the strain-level abundances as stoichiometric values for each microbe biomass reaction in the community biomass reaction. These constraints enforced that all strains grew at the experimentally measured ratios. The models were further contextualised as follows: To simulate a realistic intake of dietary nutrients in mmol per g dry weight per hour in the 108 microbiome models, an Average European diet was retrieved from the Diet Designer resource on the Virtual Metabolic Human^17^ website (https://www.vmh.life). The diet was converted to uptake fluxes through a dedicated Microbiome Modelling Toolbox function (*convertVMHDiet2AGORA.m*). Moreover, to account for host metabolism, the uptake of metabolites of host origin known to be present in the intestine (e.g., primary bile acids, host glycans) were allowed using standard COBRA Toolbox functions^52^. Finally, to simulate a realistic turnover of microbial biomass, the allowed flux through the community biomass reaction was set to be between 0.4 and 1 (mmol/person/day), corresponding to a faecal emptying of once every three days to once a day.

### Prediction of metabolic profiles

Absolute reaction presence and reaction abundances on the total community, phylum, and genus level were calculated in MATLAB using dedicated Microbiome Modelling Toolbox functions (*calculateReactionPresence.m*, *calculateReactionAbundance.m*). The computation of the total community metabolite production potential, total community metabolite uptake potential, and the contribution of each strain to each metabolite was performed in Julia v0.6.4 (https://julialang.org) using the Julia implementation of flux balance analysis, COBRA.jl^53^. COBRA.jl was performed on a high-performance cluster using the IBM CPLEX solver (IBM, Inc.) through the CPLEX interface for Julia. A customised Julia script was used that retrieved all dietary exchange reactions, faecal secretion reactions, and strain-specific internal exchange reactions for each microbiome model. This resulted on average in 13,677 exchange reactions per microbiome model, which were then each minimised and maximised using distributed flux balance analysis^53^. The fluxes were exported from Julia using the customised Julia script and further analysed in MATLAB.

### Statistical analysis

Wilcoxon rank sum test, and correction for false discovery rate (FDR) were performed in MATLAB using the ranksum and mafdr functions, respectively. Correlations between computed fluxes and species abundances were calculated using a dedicated Microbiome Modelling Toolbox function (*correlateFluxWithTaxonAbundance.m*).

The number of secreted metabolites was calculated from the computed net production fluxes (Supplementary Table 2). The association between study group and number of secreted metabolites was assessed in linear regression (ordinary least squares), introducing the log number of species as covariate. For investigating the functional dependence of the number of secreted metabolites fractional polynomials were used. A logarithmic dependency showed a significant better fit (*p* < 0.01) than a linear function and was therefore used. Next, we tested the secretion (binary: yes vs. no) on association with the study group using Fisher’s exact tests controlling for the FDR as before. The number of tests to control for was determined by counting the number of independent dichotomised secretions.

In a third step, we counted the number of sulfur-containing metabolites independently secreted for each microbiome. We tested this number on association with the study group in linear regressions. Furthermore, we explored the classification of healthy microbiomes vs. dysbiotic IBD microbiomes in logistic regression by the number and calculated the area under the curve (AUC) as metric of classification accuracy. Then, we analysed the pattern of final breakdown products of sulfur metabolism (hydrogen, sulfide, sulfate, methanethiol, sulfite, and thiosulfate) across the three study groups via Fisher’s exact test. Finally, we checked whether the presence of any glycan-degrading species statistically mediated the effects of the study group variable via the Sobel Goodman test, deriving the confidence intervals by non-parametric bootstrapping with 1000 replications^54^. These analyses were performed in STATA 14\MP (Stata Inc., College Station, USA).

### Random forests analysis

Random forests analysis was performed using the online implementation of MetaboAnalyst 5.0^31^ (https://www.metaboanalyst.ca), which relies on the MetaboAnalystR^55^ package. Briefly, the random forests classifier is built using a customisable number of trees with one-third of the data left out of the bootstrap sampling process. The left-out data is then used to estimate the out-of-bag (OOB) error. Metabolite secretion fluxes were imported through the Statistical Analysis module in the MetaboAnalyst interface without performing normalisation, transformation, or scaling of the data. The classifier was built with 5000 trees and the resulting visualisation of features ranked by their contributions to classification accuracy was exported.

### Visualisation

Phylum to amino acid and subsystem contributions were visualised using the online implementation of Circos^56^ (http://circos.ca). All other data was visualised in MATLAB version R2018b and in R version 3.5.3^57^ (https://www.r-project.org).

### Reporting summary

Further information on research design is available in the Nature Research Reporting Summary linked to this article.

## Supplementary information

Supplementary Information

Supplementary Data 1

Reporting Summary

## Data Availability

The AGORA resource^18^ is freely available at the Virtual Metabolic Human^17^ website (https://vmh.life). The relative strain abundances mapped to AGORA as performed previously^20^ as well as the models and fluxes generated in this study have been deposited at https://www.thielelab.eu/in-silico-models.

## Code availability

The COBRA Toolbox^52^, the Microbiome Modelling Toolbox^14^, and COBRA.jl^53^ are freely available at GitHub (https://github.com/opencobra). Code to reproduce the analysis is available at https://github.com/ThieleLab/CodeBase and relies on MATLAB, Julia, the COBRA Toolbox, and R.
