# Supplementary material for: Metabolic modelling reveals broad changes in gut microbial metabolism in inflammatory bowel disease patients with dysbiosis
Source: NPJ Syst Biol Appl. 2021 May 6;7:19. doi: 10.1038/s41540-021-00178-6 (PMC8102608; doi:10.1038/s41540-021-00178-6)
Supplement: Supplementary file 1 — Supplementary Information [file 41540_2021_178_MOESM1_ESM.pdf]

Supplemental material for:

Metabolic modelling reveals broad changes in gut microbial metabolism in inflammatory bowel disease patients with dysbiosis

Almut Heinken[1,2], Johannes Hertel[1,2,3], and Ines Thiele[1,2,4,5*]

[1] School of Medicine, National University of Ireland, Galway, University Road, Galway, Ireland

[2] Ryan Institute, National University of Ireland, Galway, Galway, Ireland

[3] Department of Psychiatry and Psychotherapy, University Medicine Greifswald, Greifswald, Germany

[4] Division of Microbiology, National University of Galway, Galway, Ireland

[5] APC Microbiome Ireland, Cork, Ireland

*Corresponding author: Ines Thiele, School of Medicine, National University of Ireland, Galway, University Road, Galway, Ireland. Email: ines.thiele@nuigalway.ie

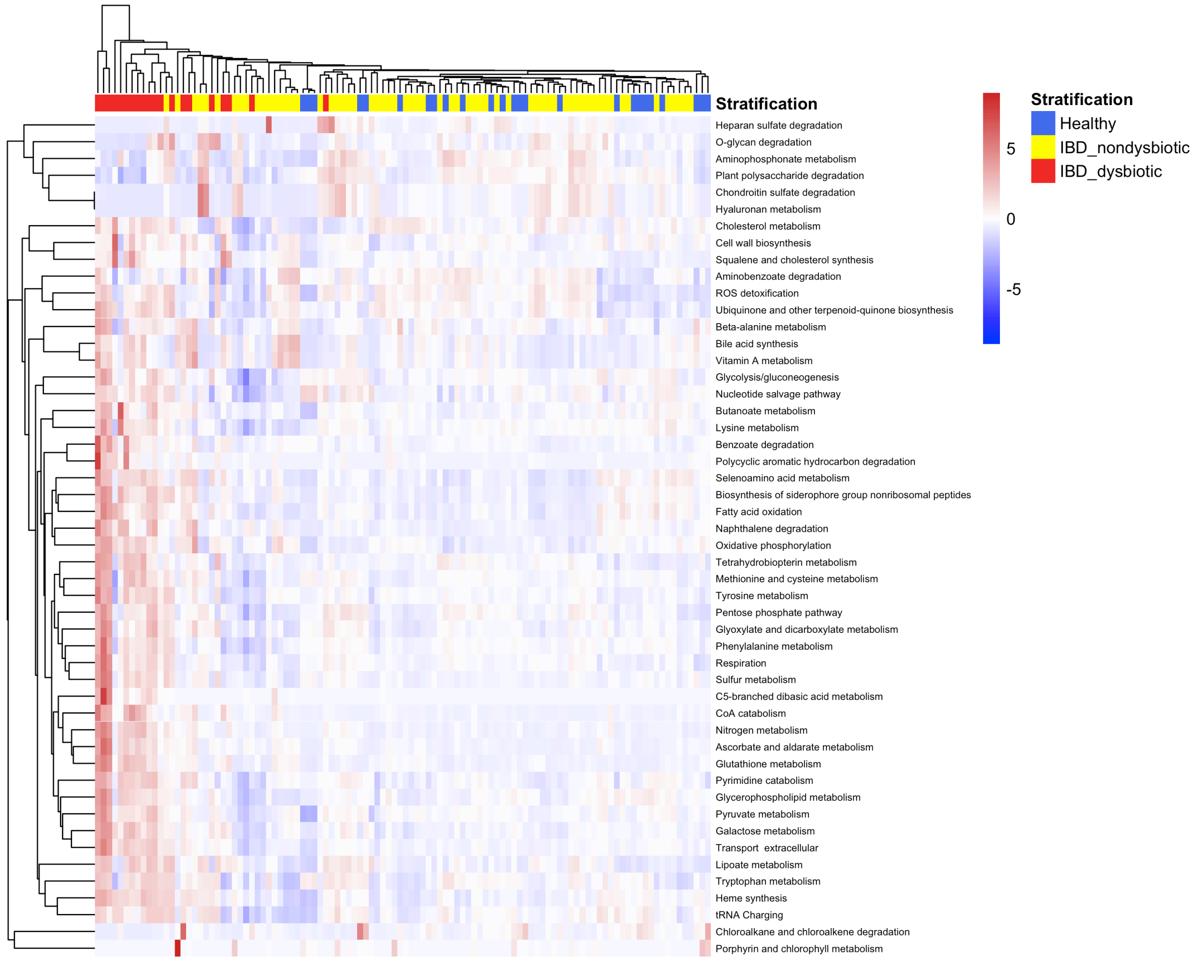

Supplementary Figure 1: Quantitative abundances of pathways in the 108 microbiomes. Shown are all subsystems that, after summarizing abundances for the corresponding reactions, were significantly different between healthy and IBD and/or between the dysbiotic and non-dysbiotic IBD cluster after correcting for false discovery rate.

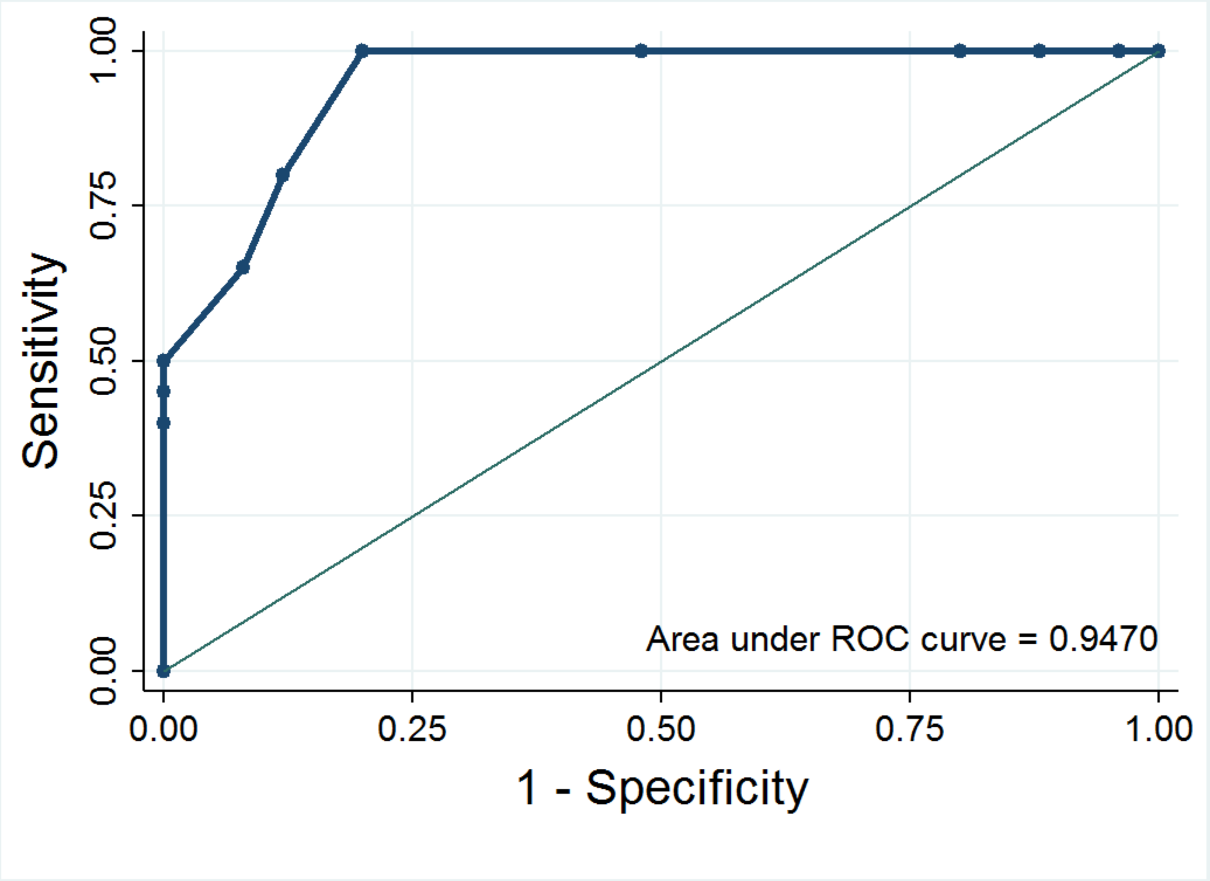

Supplementary Figure 2: AUC from logistic regression, classifying healthy individuals using the number of independently secreted sulphur metabolites as only predictor. AUC is significantly bigger than 0.5, p=0.003.

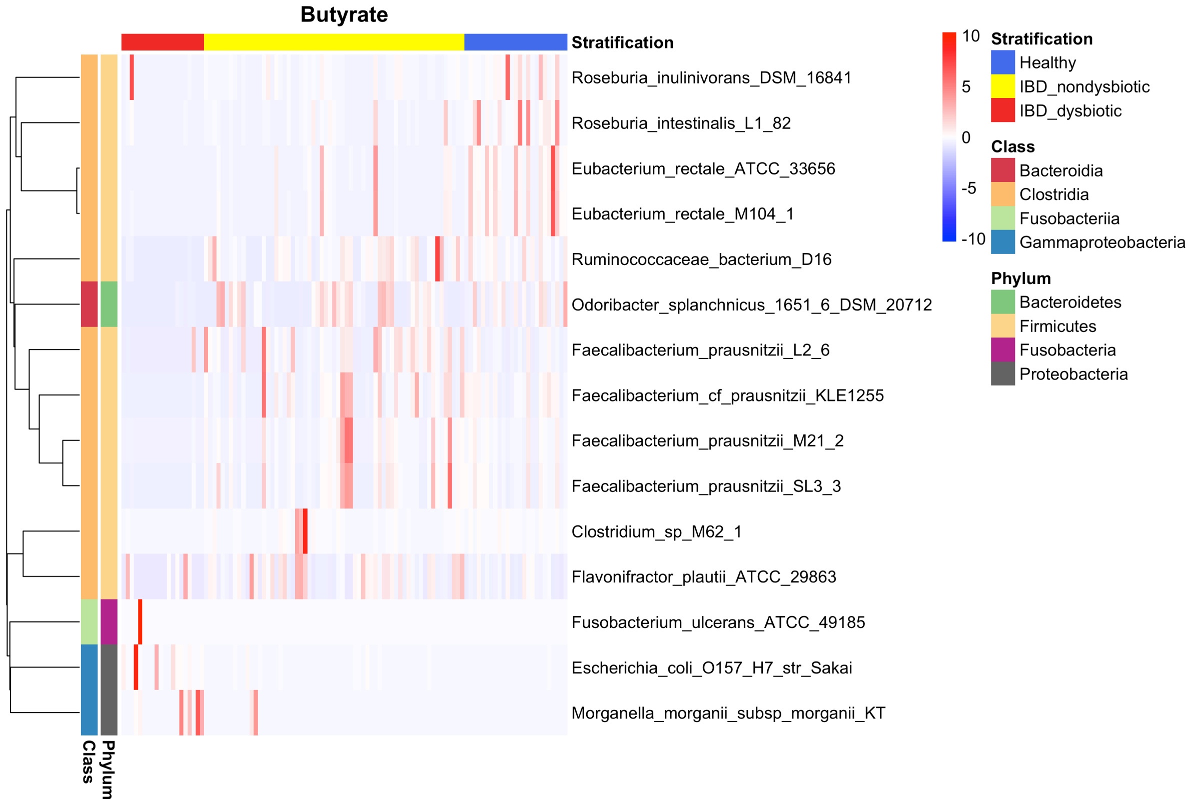

Supplementary Figure 3: Strain-level contributions to total butyrate production (mmol/person/day) in all 108 personalised microbiome models.

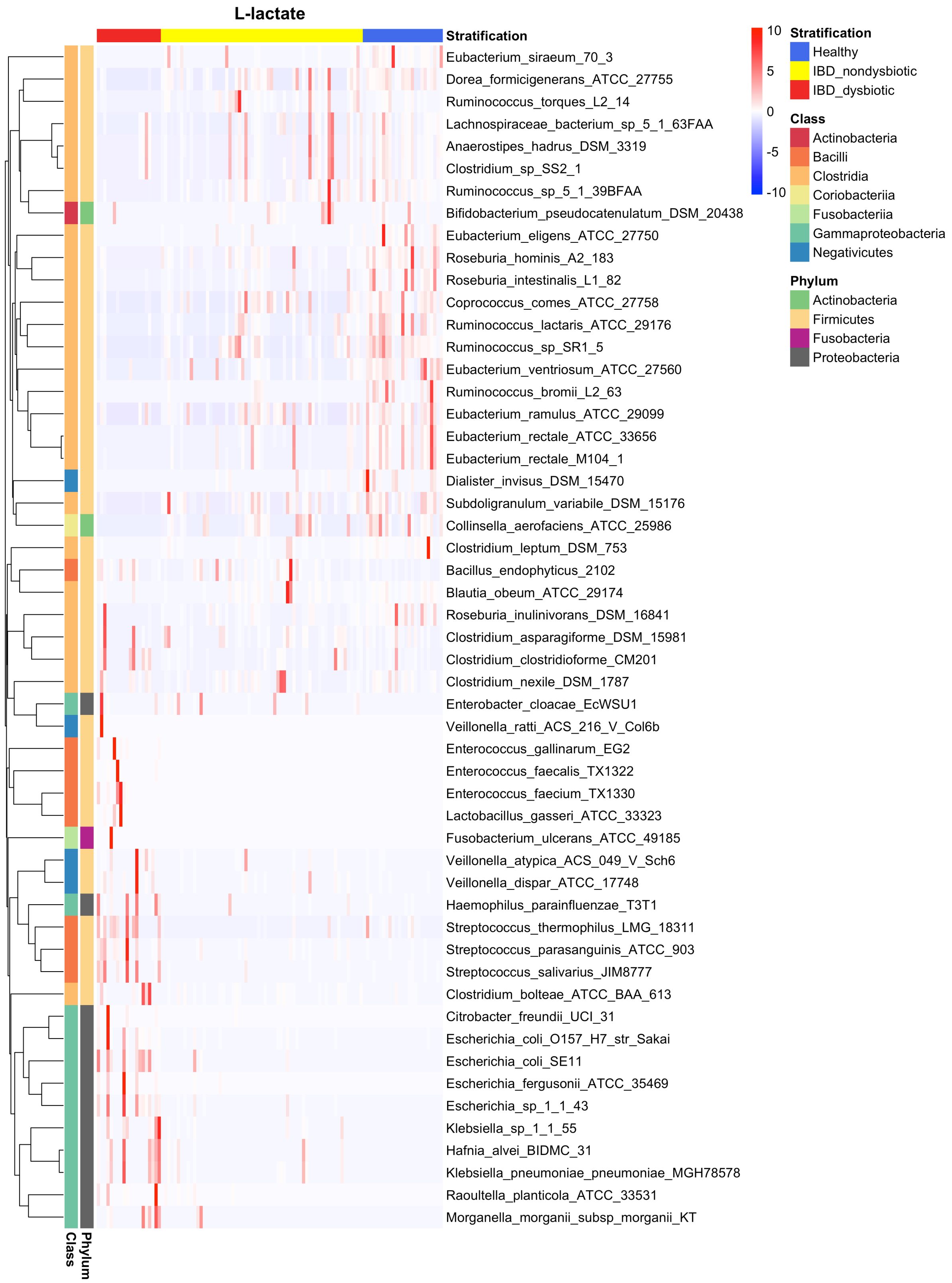

Supplementary Figure 4: Strain-level contributions to total L-lactate production (mmol/person/day) in all 108 personalised microbiome models.

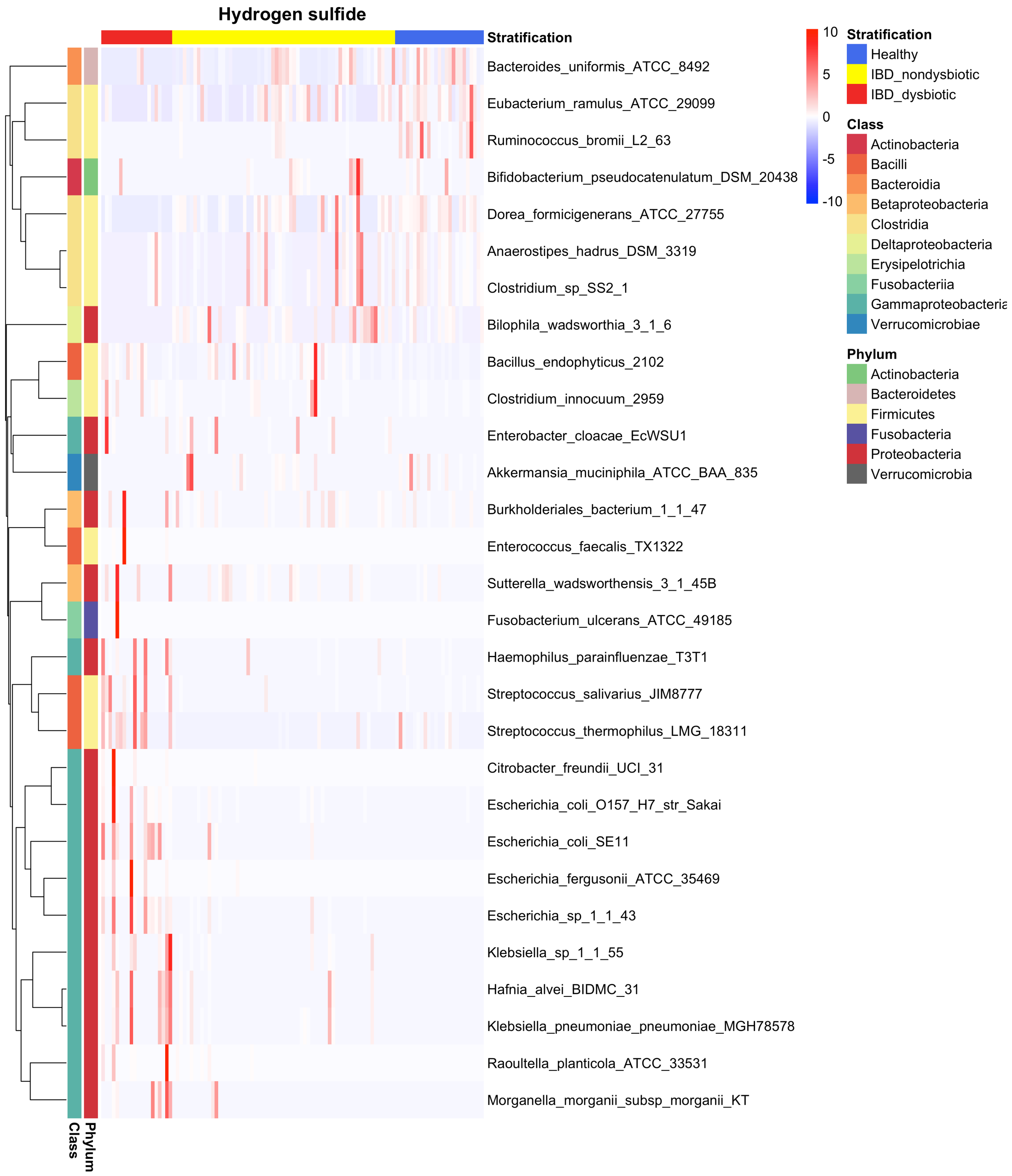

Supplementary Figure 5: Strain-level contributions to total hydrogen sulphide production (mmol/person/day) in all 108 personalised microbiome models.

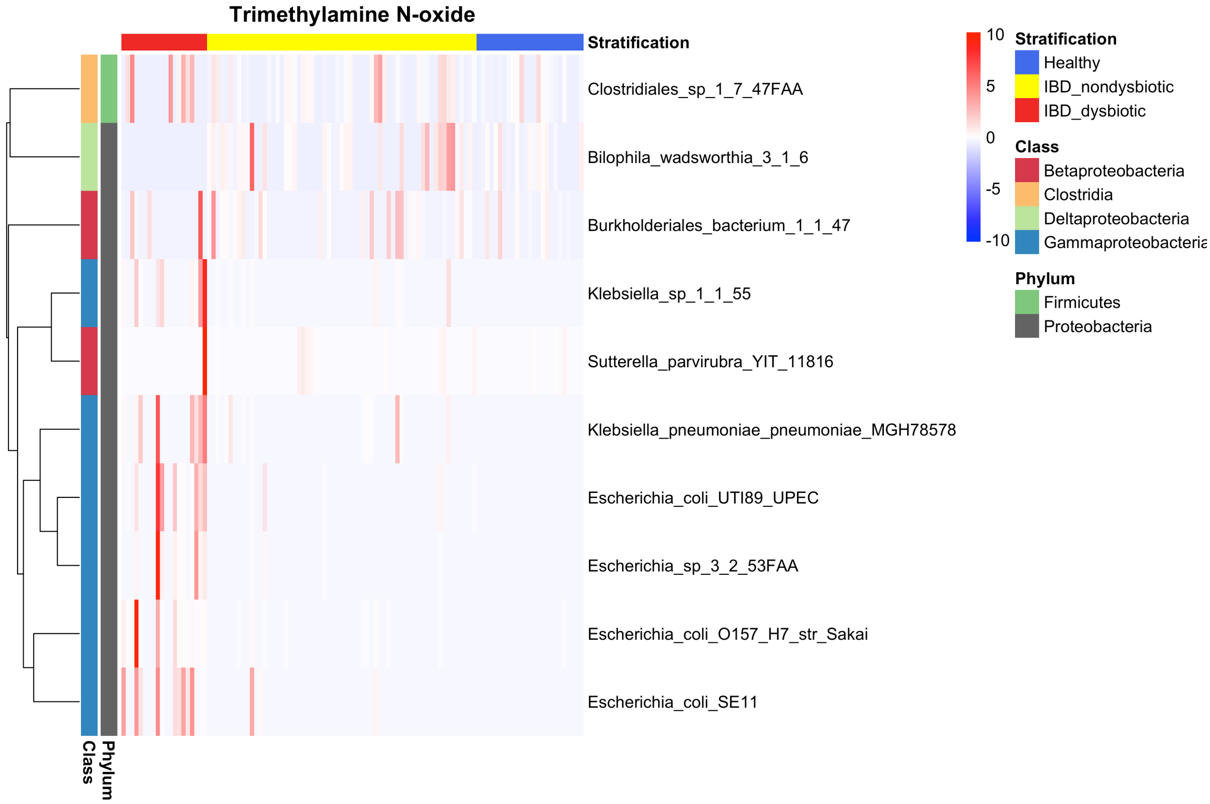

Supplementary Figure 6: Strain-level contributions to total trimethylamine-N-oxide production (mmol/person/day) in all 108 personalised microbiome models.

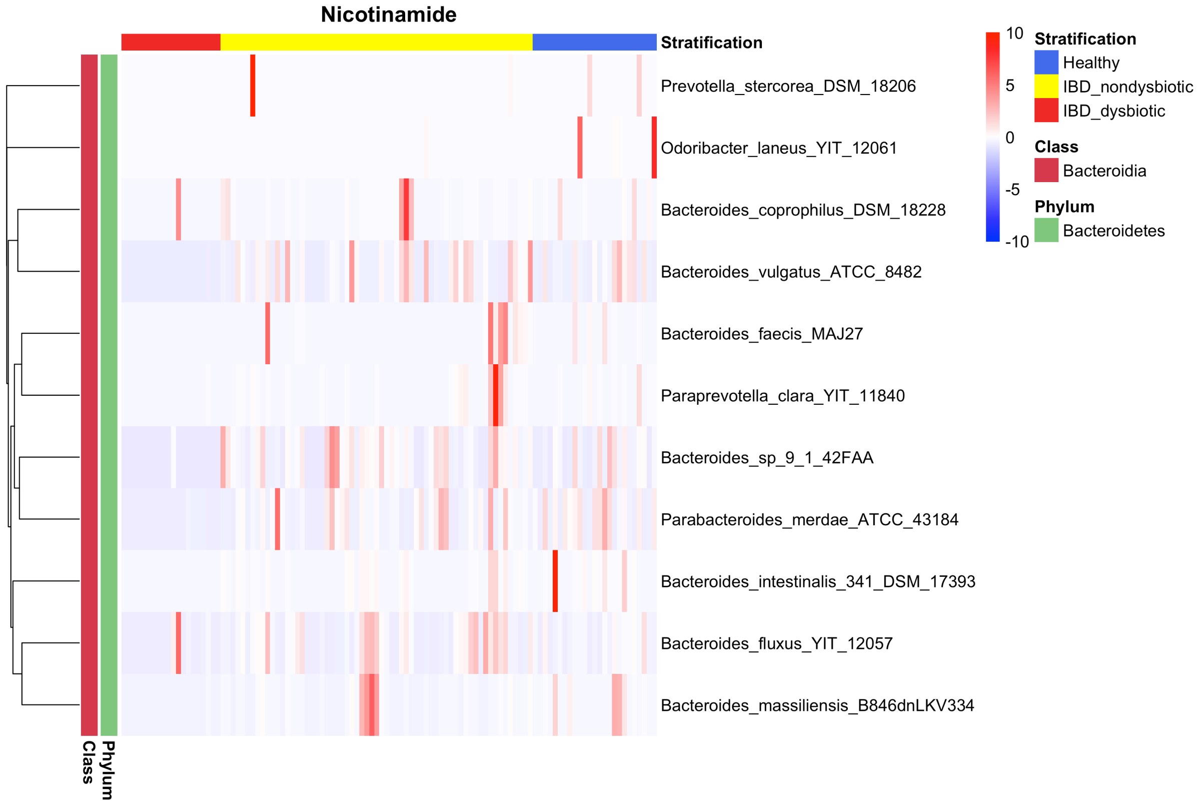

Supplementary Figure 7: Strain-level contributions to total nicotinamide production (mmol/person/day) in all 108 personalised microbiome models.

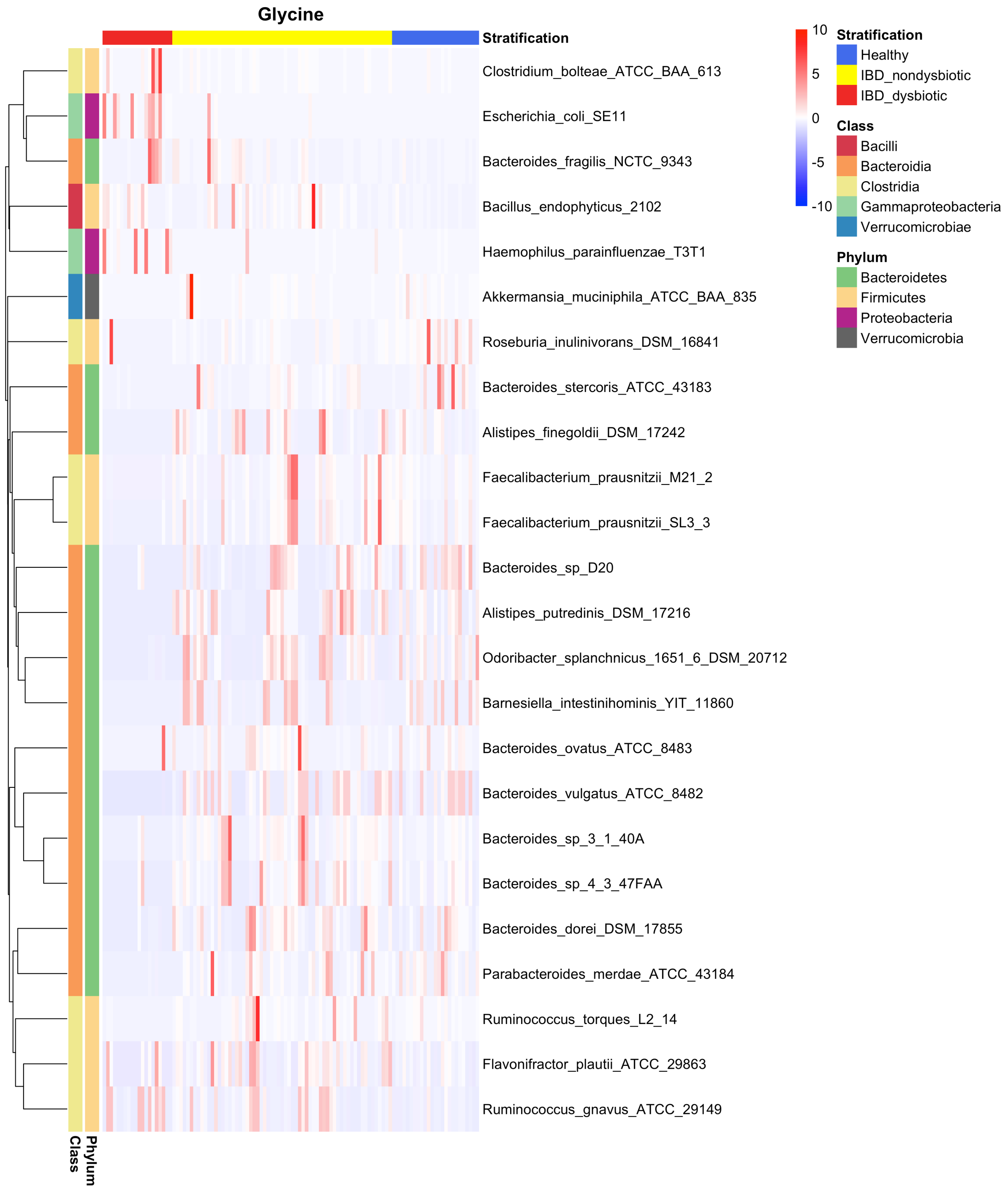

Supplementary Figure 8: Strain-level contributions to total glycine production (mmol/person/day) in all 108 personalised microbiome models.

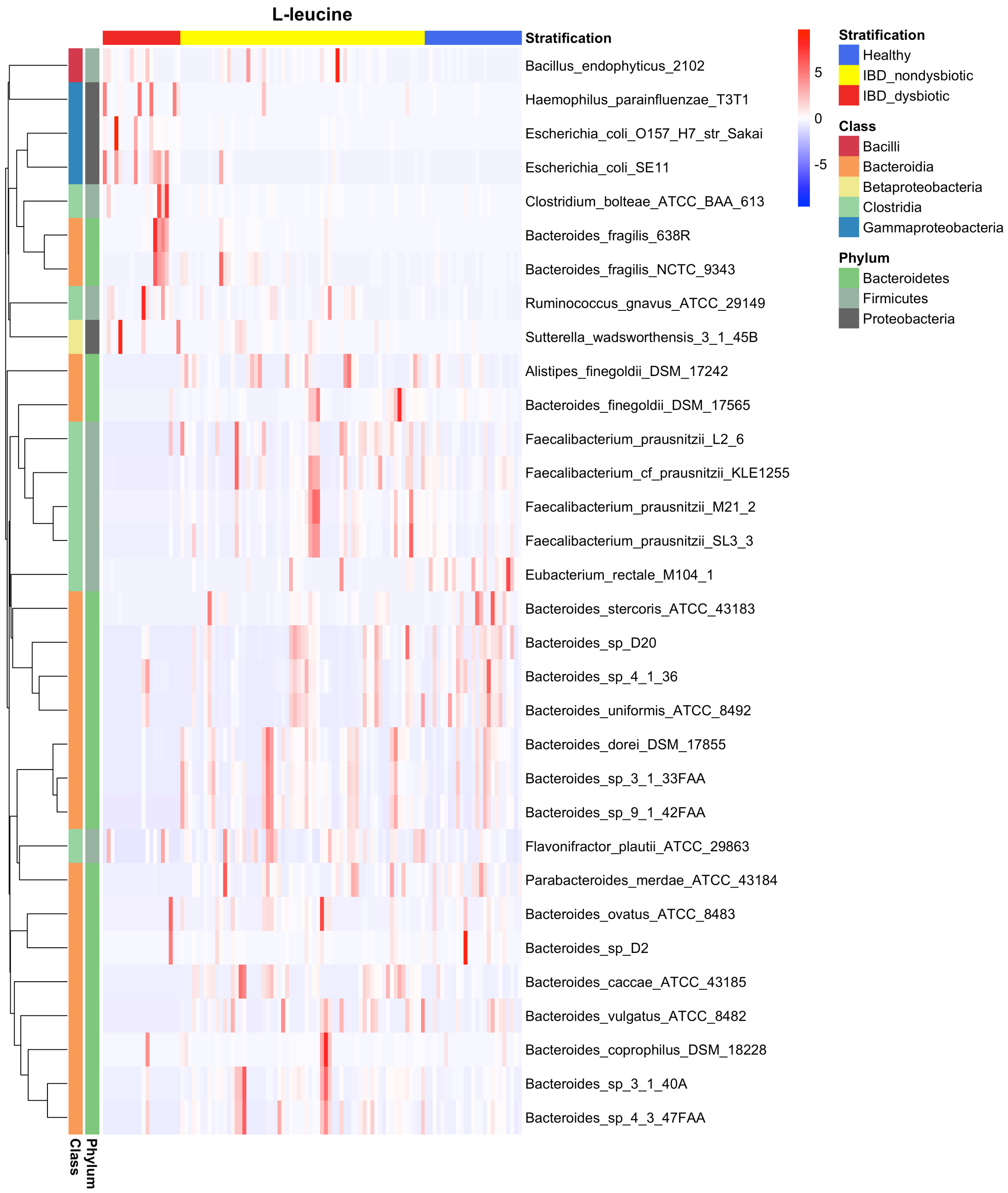

Supplementary Figure 9: Strain-level contributions to total L-leucine production (mmol/person/day) in all 108 personalised microbiome models.

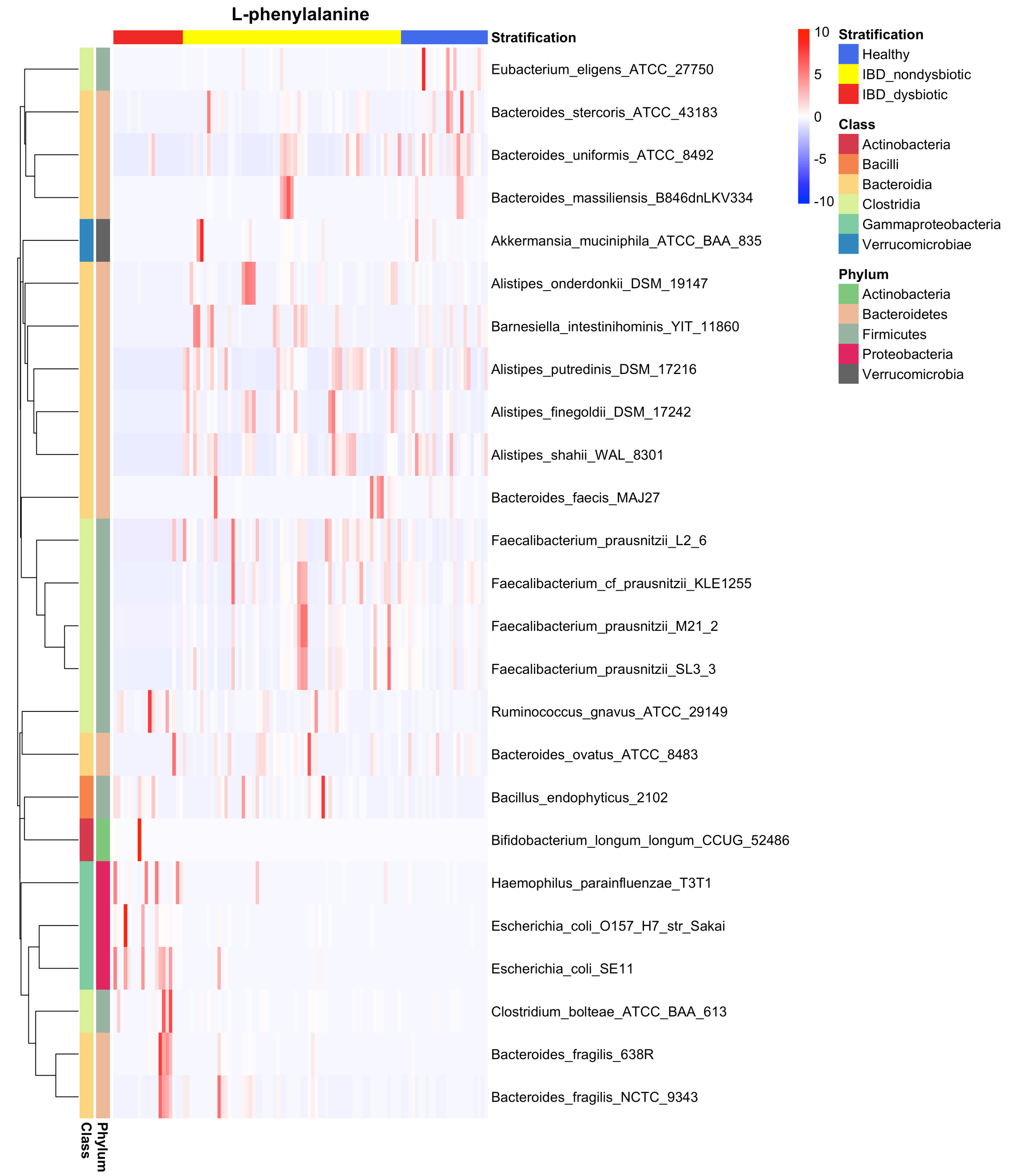

Supplementary Figure 10: Strain-level contributions to total L-phenylalanine production (mmol/person/day) in all 108 personalised microbiome models.

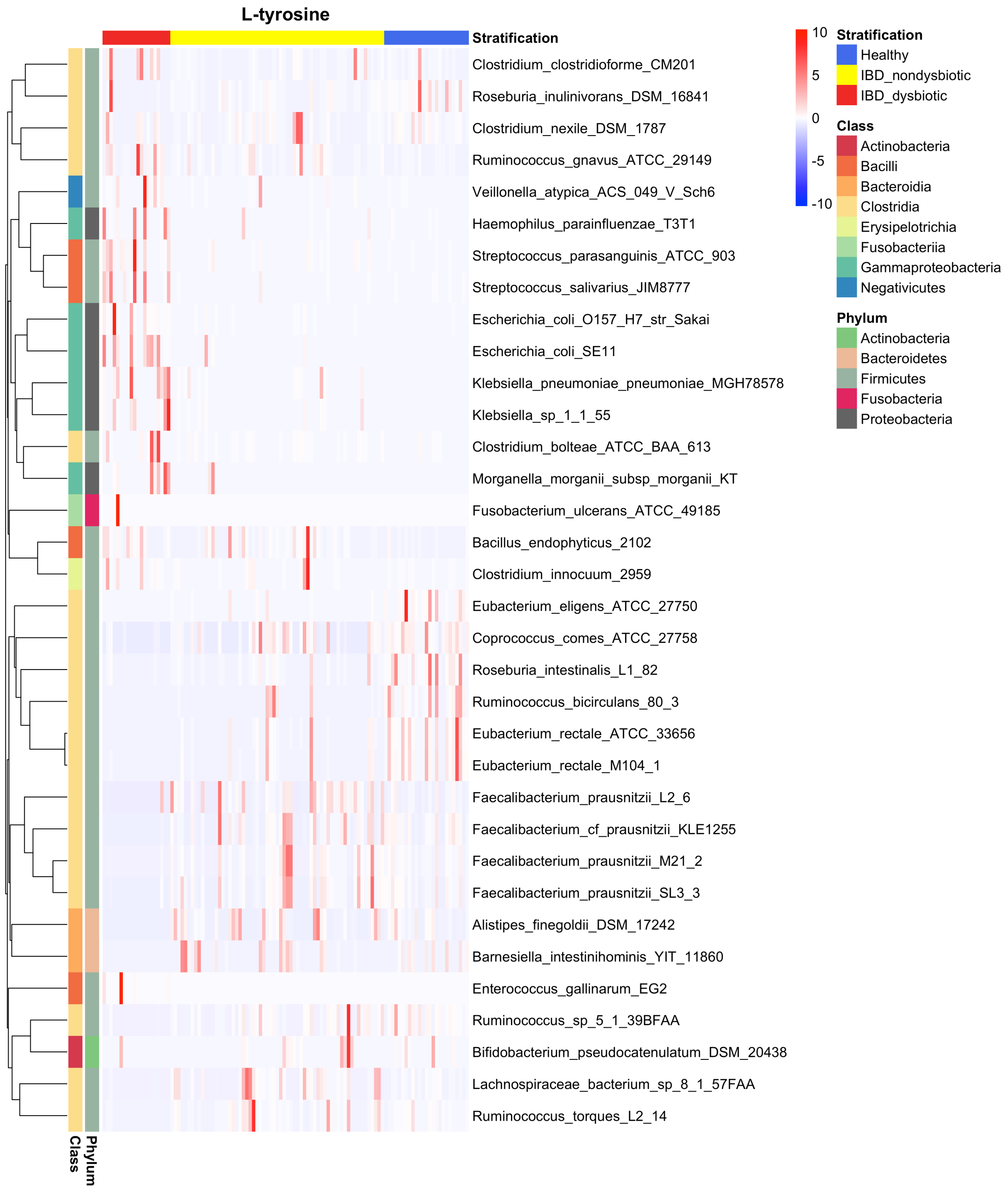

Supplementary Figure 11: Strain-level contributions to total L-tyrosine production (mmol/person/day) in all 108 personalised microbiome models.

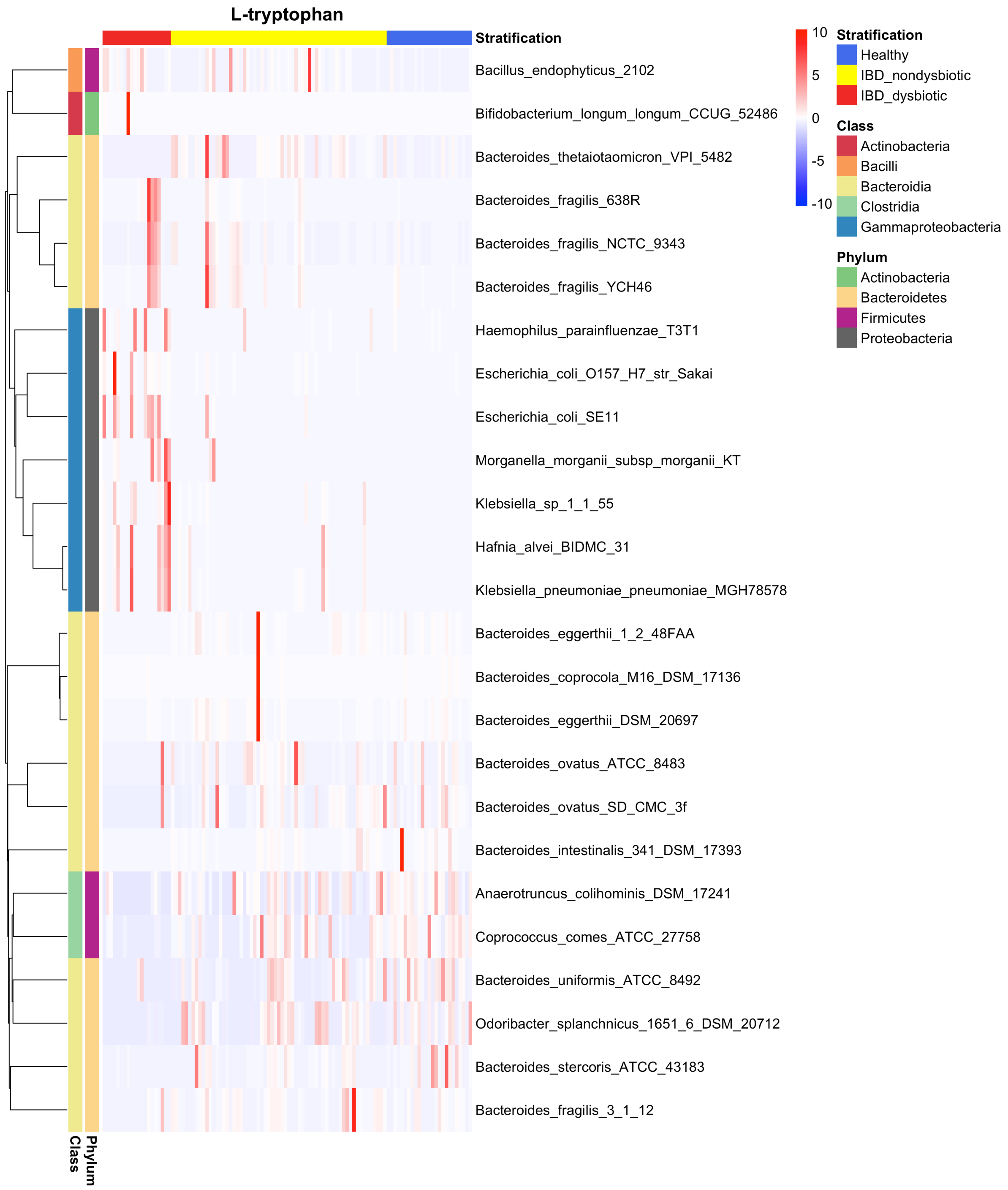

Supplementary Figure 12: Strain-level contributions to total L-tryptophan production (mmol/person/day) in all 108 personalised microbiome models.
